# The efficacy and safety of kappa opioid receptor (KOR) agonists in patients with uraemic pruritus: a systematic review and network meta-analysis

**DOI:** 10.1093/ckj/sfaf131

**Published:** 2025-06-23

**Authors:** Hanqi Yang, Ming Pei, Jingbo Zhai, Zijun Zhou, Yunze Xing, Qiumei Lan, Yixin Zhu, Xuchen Wang, Bo Yang

**Affiliations:** Department of Nephrology, First Teaching Hospital of Tianjin University of Traditional Chinese Medicine, Tianjin, China; Department of Nephrology, National Clinical Research Center for Chinese Medicine Acupuncture and Moxibustion, Tianjin, China; Department of Nephrology, First Teaching Hospital of Tianjin University of Traditional Chinese Medicine, Tianjin, China; Department of Nephrology, National Clinical Research Center for Chinese Medicine Acupuncture and Moxibustion, Tianjin, China; School of Public Health, Tianjin University of Traditional Chinese Medicine, Tianjin, China; Department of Nephrology, First Teaching Hospital of Tianjin University of Traditional Chinese Medicine, Tianjin, China; Department of Nephrology, National Clinical Research Center for Chinese Medicine Acupuncture and Moxibustion, Tianjin, China; Department of Nephrology, First Teaching Hospital of Tianjin University of Traditional Chinese Medicine, Tianjin, China; Department of Nephrology, National Clinical Research Center for Chinese Medicine Acupuncture and Moxibustion, Tianjin, China; Department of Nephrology, Third Affiliated Hospital of Guangxi University of Traditional Chinese Medicine, Liuzhou Chinese Medicine Hospital, Liuzhou, China; Department of Nephrology, First Teaching Hospital of Tianjin University of Traditional Chinese Medicine, Tianjin, China; Department of Nephrology, National Clinical Research Center for Chinese Medicine Acupuncture and Moxibustion, Tianjin, China; Department of Nephrology, First Teaching Hospital of Tianjin University of Traditional Chinese Medicine, Tianjin, China; Department of Nephrology, National Clinical Research Center for Chinese Medicine Acupuncture and Moxibustion, Tianjin, China; Department of Nephrology, First Teaching Hospital of Tianjin University of Traditional Chinese Medicine, Tianjin, China; Department of Nephrology, National Clinical Research Center for Chinese Medicine Acupuncture and Moxibustion, Tianjin, China

**Keywords:** kappa opioid receptor, network meta-analysis, uraemic pruritus

## Abstract

**Background:**

Uraemic pruritus (UP) is an increasingly significant health burden. However, current treatments are often unsatisfactory and associated with numerous adverse reactions. Recently, several large randomized controlled trials (RCTs) have confirmed that kappa opioid receptor (KOR) agonists, which target the endogenous opioid system, are effective in controlling symptoms. We compared the efficacy and safety of currently available KOR agonists for the treatment of UP.

**Methods:**

We conducted a systematic review and network meta-analysis (NMA) of RCTs to assess the efficacy and safety of KOR agonists in patients with UP. The primary outcomes were pruritus-related scales and adverse events. Two independent reviewers evaluated RCTs for eligibility and extracted relevant data, with discrepancies resolved by consensus or a third reviewer. We utilized a fixed effects model within a Bayesian framework for the NMA. Dichotomous variables were presented as risk ratios (RRs) and continuous variables were merged using standardized mean differences. Statistical analyses were performed using R 4.2.3 and JAGS 4.3.0. The risk of bias was assessed using the RoB 2 tool and the certainty of findings was rated according to Grading of Recommendations Assessment, Development and Evaluation criteria. The study protocol was registered on PROSPERO (CRD42020169955).

**Results:**

Ten studies with 2483 participants were included. Concerning the primary endpoints, difelikefalin at doses of 0.25 µg/kg, 0.5 µg/kg, 1.0 µg/kg and 1.5 µg/kg, nalfurafine at 2.5 µg and 5 µg and nalbuphine at 120 mg were significantly effective in reducing itching severity compared with placebo. For the secondary endpoint, all four doses of difelikefalin were associated with higher rates of adverse events compared with placebo, while other interventions showed rates comparable to those of placebo and did not present statistically significant differences.

**Conclusion:**

In summary, difelikefalin at doses of 0.25 µg/kg and 0.5 µg/kg, along with nalfurafine at 0.25 µg/kg and 0.5 µg/kg, can be considered recommended therapeutic options for UP treatment.

KEY LEARNING POINTS **What was known:**

- Uraemic pruritus (UP) demonstrates wide geographical variation in prevalence (20–80%) across regions and imposes a substantial health burden.
- UP is associated with adverse health outcomes.
- With the emergence of the kappa opioid receptor (KOR) agonists that target the endogenous opioid system as potential treatments of UP.

**This study adds:**

- This study represents the first network meta-analysis comparing the efficacy and safety of currently available KOR agonists for the treatment of UP.
- Some doses of KOR were significantly better than placebo in relieving itching.
- Difelikefalin at doses of 0.25 µg/kg and 0.5 µg/kg, along with nalfurafine at doses of 0.25 µg/kg and 0.5 µg/kg, are recommended as the preferred choice in clinical practice.

**Potential impact:**

- When designing future trials, longer follow-up periods should be considered to capture the potential recurrence of symptoms and to provide a more comprehensive understanding of the long-term benefits and risks associated with KOR agonist therapy.
- More head-to-head trials are warranted to confirm these findings.

## INTRODUCTION

Uraemic pruritus (UP) is a prevalent and relapsing condition in patients with end-stage renal disease that significantly impacts quality of life [1, 2]. Studies have indicated that the prevalence of UP varies significantly across different countries, dialysis methods and study populations, with the reported rates ranging from 20% to 80% [3–6]. Although the prevalence of severe pruritus has decreased over the past 2 decades, the burden of pruritus on chronic kidney disease (CKD) patients remains underestimated in many regions [7, 8].

UP is associated with various adverse health outcomes, including sleep disturbances, fatigue, anxiety and depression, as well as skin lesions and secondary infections caused by repetitive scratching behaviour secondary to pruritus [9, 10]. Increasing itch severity is also found to be closely related to the longer recovery time after a haemodialysis (HD) session [11], both of which can reduce physical and mental quality of life [11–13]. Furthermore, there is a significant correlation between UP and increased mortality, and the mechanism may involve a multifactorial combination of itch-induced disruption of the sleep–wake cycle, exacerbation of chronic inflammation and an increased risk of secondary infections [6, 14, 15].

At present, treatments for UP include topical therapies (e.g. emollients and capsaicin cream), systemic agents (e.g. antihistamines and gabapentinoids) and non-pharmacological interventions (e.g. phototherapy and acupuncture) as adjuvant options. Although various treatment options are available, they are associated with certain limitations, such as limited efficacy and significant side effects [16, 17]. As for clinical studies, even though preferential results for some drugs have been reported, these were usually followed by studies with contradictory results. For instance, a systematic review of two randomized controlled trials (RCTs) found that ondansetron provided no benefit over placebo in the treatment of UP [18]. Oral antidepressants can cause neurological side effects, including mild fatigue and drowsiness. Furthermore, immunomodulators and neuromodulators like thalidomide may alleviate chronic refractory pruritus but they are also associated with numerous adverse effects, including teratogenicity, peripheral neuropathy, sedation, dizziness and thromboembolism. As a result, no universally accepted first-line therapy is available at present. In this regard, uraemic pruritus is still a very difficult disease to treat and new effective treatments are needed.

The emergence of the kappa opioid receptor (KOR) agonists, which target the endogenous opioid system, may provide new potential treatments for UP [19]. As suggested in some studies, pruritus might result from the antagonism of peripheral KOR or an imbalance between the stimulation and antagonism of μ-opioid receptors and KOR [20, 21]. KOR agonists exert dual antipruritic mechanisms. Peripherally it can block pruritogen release by targeting κ-opioid receptors on cutaneous keratinocytes, sensory nerves and immune cells. Centrally, they work via spinal cord receptor modulation of thalamic transmission and cortical disruption of pruriceptive signal integration [2, 22–26]. Recently, several large RCTs investigating KOR agonists for the treatment of UP have shown promising results. For example, the KOR agonist difelikefalin, which is already on the market, has demonstrated significant efficacy in the treatment of UP, with a relatively acceptable safety profile. However, due to the lack of direct comparison, which KOR agonists offer the greatest benefit for patients with UP remains elusive, as few studies have analysed or ranked the efficacy and safety of the existing KOR agonists in treating UP. To address this issue, we employed a systematic review with Bayesian network meta-analysis (NMA) to enable both dose-stratified direct comparisons of KOR agonists and indirect comparative efficacy evaluations across different KOR agonists [27].

## MATERIALS AND METHODS

We conducted this NMA according to the Preferred Reporting Items for Systematic Reviews and Meta-Analyses [28] and extension statement for NMAs. The study protocol was registered on PROSPERO (CRD42020169955).

### Search strategies

We identified studies from PubMed, Embase, Web of Science, Cochrane Central Register of Controlled Trials and Web of Science without time and language restrictions from inception until March 2024. The search terms included ‘puritus’, ‘uremia’ and ‘kappa opioid receptor agonist’. Furthermore, a manual search of the reference lists of pertinent articles, reviews and meta-analyses was conducted to preclude the omission of any significant literature. Moreover, ClinicalTrials.gov and the Chinese Clinical Trial Registry were also be searched to identify potential grey literature. The detailed search strategies are available in Supplementary Table S1.

### Study selection

Using the inclusion and exclusion criteria outlined below, two reviewers independently selected eligible studies by screening titles and abstracts and then assessed the full text. Any discrepancies between the reviewers were resolved through discussion or by consulting a third investigator. We included RCTs involving patients with UP, regardless of age, gender, race or nationality. Quasi-RCTs, cross-over trials, abstracts and letters were excluded. We included RCTs comparing a KOR agonist with either a placebo or another KOR agonist. There were no restrictions on dosage, treatment duration, type of agonist or blinding status. RCTs employing KOR agonist combined with another active treatment were excluded. RCTs had to report at least one of the following outcomes: the primary outcome measures included the changes from baseline in the visual analogue scale (VAS), the Worst Itch Numeric Rating Scale (WINRS), the Skindex-10 and the 5-D itch scale. The secondary outcome measure was the incidence of adverse events.

### Data extraction

A data extraction form was developed and piloted. The extracted data included the first author, publication year, sample size, patient age, intervention measures, outcome indicators, treatment duration and adverse events. Data extraction was performed by one reviewer and verified by a second reviewer. Discrepancies were resolved by consultation with other members of the team.

### Quality assessment

The risk of bias in the included RCTs was evaluated using the revised Cochrane risk of bias tool (RoB 2.0). This comprehensive tool evaluates potential biases arising from five key domains: randomization process, deviations from intended interventions, missing outcome data, measurement of the outcome and selection of the reported outcome. The quality of evidence for any significant outcomes in the NMA was scored according to the Grading of Recommendations Assessment, Development and Evaluation (GRADE) process. Two evaluators independently conducted the risk of bias assessments and the quality of evidence evaluations. Any discrepancies were resolved through discussion.

### Statistical analysis

A NMA was conducted within a Bayesian framework that integrated both direct and indirect estimates to derive a comprehensive network effect. To be specific, direct estimates were obtained from head-to-head comparisons, while indirect estimates were calculated based on data pooled through a common comparator, such as placebo or non-treatment. Four Markov chains were employed, each with an initial value of 2.5, a pre-iteration count of 5000 and a total of 25 000 iterations for updating. For continuous outcomes, changes before and after treatment were assessed and standardized mean differences (SMDs) were used to pool the effect sizes, since some results were reported in the form of means, whereas others were least square means (LSMs). Dichotomous outcomes were described as risk ratios (RRs) with corresponding 95% confidence intervals (CIs). To evaluate the relative effectiveness of interventions, the surface under the cumulative ranking curve (SUCRA), which ranks the probability of each intervention being the most effective, was utilized. All statistical analyses were conducted using JAGS version 4.3.0 and R version 4.2.3 (R Foundation for Statistical Computing, Vienna, Austria).

## RESULTS

### Literature selection

**Figure 1:**
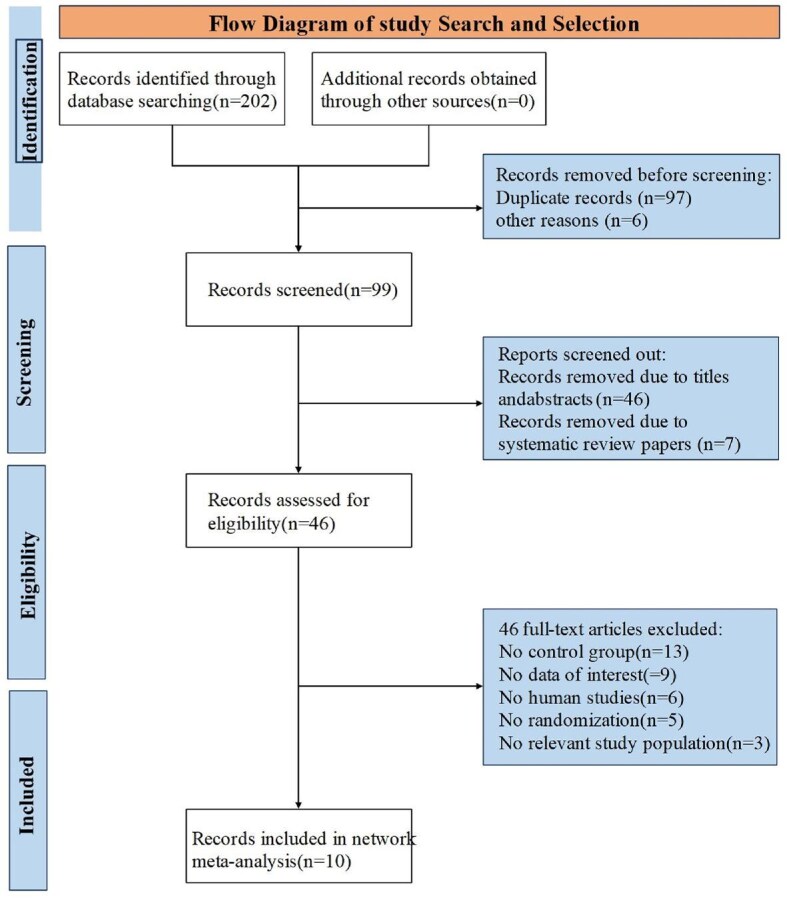
Flow diagram of study search and selection.

Potentially eligible studies were collected from the comprehensive literature search and imported into EndNote software (Clarivate, London, UK) to remove duplicate studies. Then, two reviewers independently deleted ineligible studies by checking titles and abstracts according to the inclusion and exclusion criteria. Full texts of the remaining studies were read to identify included studies. Disagreements were handled in consultation with a third reviewer. Eventually, 10 eligible studies were included in the final statistical analysis. The study search and selection process are illustrated in Fig. 1.

### Study characteristics

The characteristics of the 10 RCTs are summarized in Table 1. All studies were published between 2005 and 2023, with participant counts for each trial ranging from 45 to 471 subjects. The total number of participants across all trials was 2483. Among these studies, five trials involving 1528 participants compared difelikefalin with a placebo [29–32], while four trials with 574 participants evaluated nalfurafine against a placebo [33–35]. Additionally, one study with 371 participants assessed nalbuphine compared with a placebo [36]. Of the included studies, five reported outcomes using the numerical rating scale [29–31, 36], four reported outcomes using the VAS [33–35], seven used the Skindex-10 and six used the 5-D scale [29–32].

**Table 1:** Basic characteristics of the included studies.

Study	Publish year	Country	Study design	Treatment and follow-up periods	Intervention	Sample size	Age (years), mean (SD)	Pruritus assessment tool	Adverse events
Narita *et al*. [30]	2022	Japan	RCT	8-week treatment,2-week follow-up periods	Placebo	63	64.1 (12.7)	WINRS, Skindex-10, 5-D	42/63
					Difelikefalin 0.25 μg/kg	61	64.2 (11.2)		44/61
					Difelikefalin 0.5 μg/kg	61	65.6 (11.4)		47/61
					Difelikefalin 1.0 μg/kg	62	64.4 (11.7)		53/62
Zhang *et al*. [33]	2023	China	RCT	14-day treatment,8-day follow-up periods	Placebo	27	56.6 (14.51)	VAS	23/27
					Nalfurafine 5 μg	57	57.2 (14.33)		44/57
					Nalfurafine 2.5 μg	57	53.7 (12.35)		45/57
Yosipovitch *et al*. [29]	2023	USA	RCT	12-week treatment period	Placebo	67	65.6 (12.1)	WINRS, Skindex-10, 5-D	34/67
					Difelikefalin 1.0 μg/kg	67	67.5 (10.7)		39/67
					Difelikefalin 0.5 μg/kg	66	69 (12)		34/66
					Difelikefalin 0.25 μg/kg	69	65.7 (11)		45/69
Fishbane *et al*. [32]	2020	USA	RCT	12-week treatment period	Placebo	188	56.89 (13.9)	5-D, Skindex-10	117/188
					Difelikefalin 0.5 μg/kg	189	58.2 (11.2)		130/189
Kumagai *et al*. [34]	2010	Japan	RCT	14-day treatment period, 8-day follow-up periods	Placebo	111	59.6 (11.8)	VAS	56/111
					Nalfurafine 5 μg	114	59.6 (11.5)		71/114
					Nalfurafine 2.5 μg	112	61.0 (11.4)		55/112
Wikström *et al*. [35]	2005	Japan	RCT	4-week treatment,2-week follow-up periods	Placebo	25	None	VAS	13/25
					Nalfurafine 5 μg	26			17/26
Fishbane *et al*. [31]	2020	USA	RCT	8-week treatment,1-week follow-up periods	Placebo	45	60	WINRS, Skindex-10, 5-D	19/45
					Difelikefalin 0.5 μg/kg	44	57		37/44
					Difelikefalin 1.0 μg/kg	41	59		29/41
					Difelikefalin 1.5 μg/kg	44	56.5		34/44
Mathur *et al*. [36]	2017	USA	RCT	8-week treatment period	Placebo	123	57 (13)	WINRS, Skindex-10	none
					Nalbuphine 120 mg	120	55 (12)		none
					Nalbuphine 60 mg	128	55 (12)		none
Not mentioned	Not yet	USA	RCT	8-week treatment, 1-week follow-up period	Placebo	11	55.2 (12.94)	WINRS, VAS, Skindex-10, 5-D	6/11
					Nalfurafine 2.5 μg	10	58.5 (10.62)		6/10
					Nalfurafine 5 μg	13	54.6 (12.78)		5/13
					Nalfurafine 10 μg	11	55.3 (13.64)		9/11
Menzaghi *et al*.	Not yet	USA	RCT	12-week treatment period	Placebo	236	59.6 (13.07)	Skindex-10, 5-D	43/236
					Difelikefalin 0.5 μg/kg	235	59.7 (13.11)		56/235

### Risk of bias

**Figure 2:**
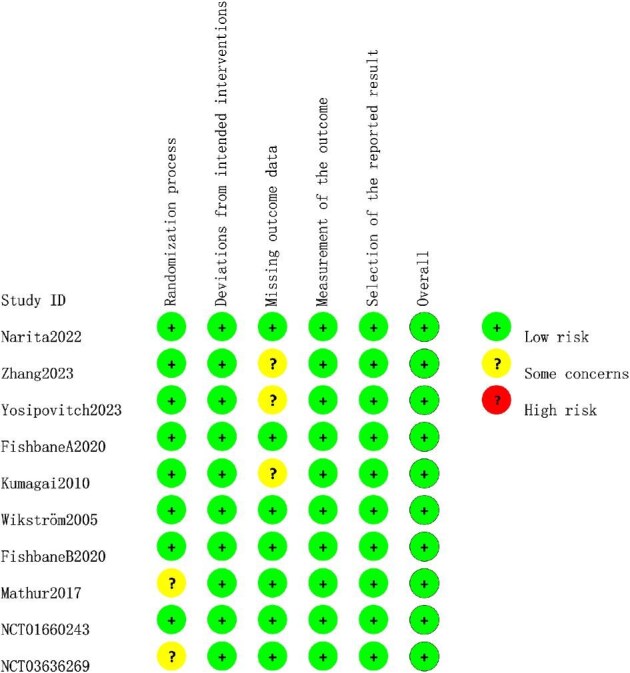
Quality assessment results of risk of bias items in included studies.

**Figure 3:**
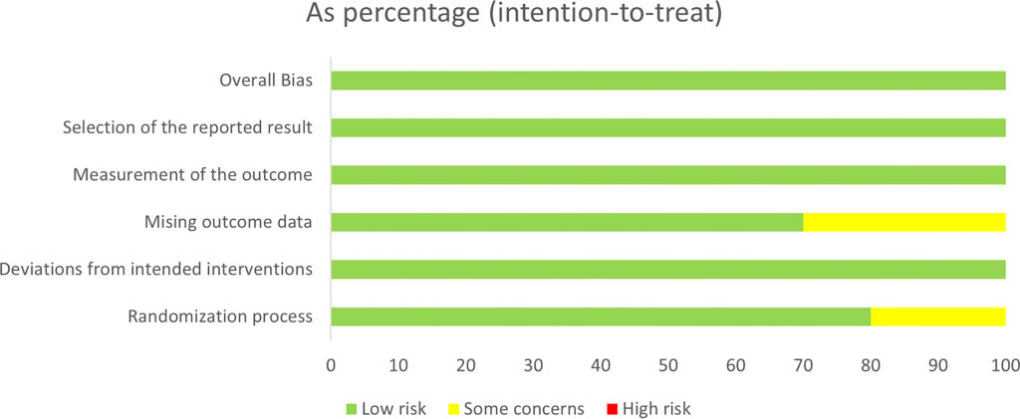
Percentages of items of included articles that produced risks of bias.

The detailed results of the bias risk assessment for all included studies are shown in Figs. 2 and 3. Overall, the risk of bias across the assessed domains was generally low or unclear. These studies compared opioid receptor treatment regimens with placebos and reported clinically confirmed outcomes. Although all studies indicated patient randomization into groups, four studies [32, 35] did not explicitly describe the method of random sequence generation. Blinding was consistently applied for both participants and staff, resulting in a low risk of bias in this domain. Most studies did not report missing data and were similarly rated as low risk. However, three studies required data conversion and were rated as having an unclear risk of bias [29, 31, 34]. No evidence of selective reporting was found and all studies were rated as having a low risk of bias in this regard. The results showed that the GRADE quality of the majority of outcomes was high or moderate. Supplementary Table S2 shows details of the GRADE assessment for each outcome.

### Network map

**Figure 4:**
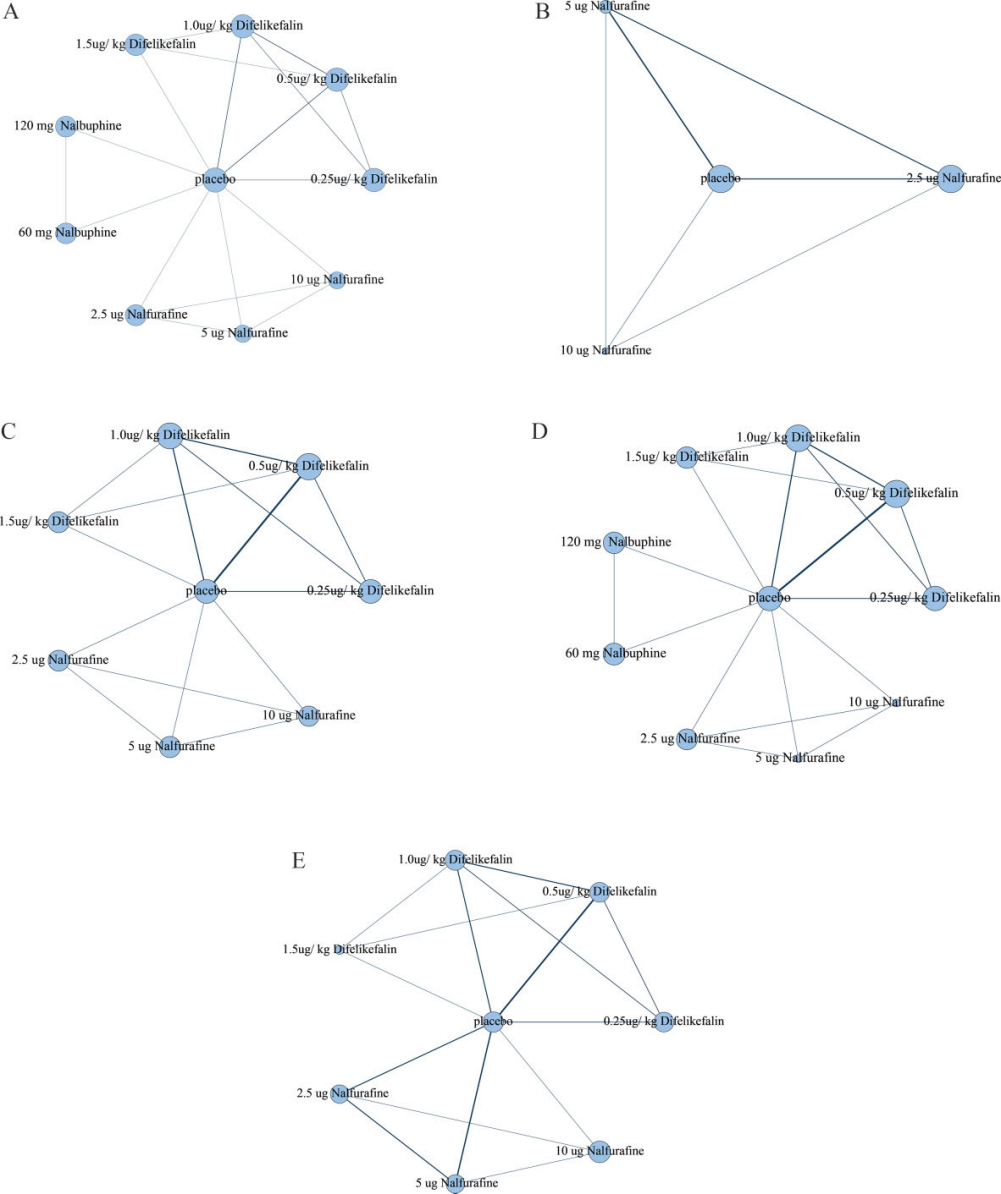
Network map of different dosages of KOR against and placebo for pruritus relief(A-D) and adverse events (E).

Network evidence for each outcome indicator was plotted to visualize the comparisons between interventions (Fig. 4) [37]. The size of each node was proportional to the number of included studies, while the edge of each comparison was weighted according to the number of patients involved in that comparison. A closed triangular formation among a subset of nodes within the network indicated the presence of both direct and indirect comparative analyses conducted concurrently among certain interventions.

### The WINRS

As shown in Table 1, five RCTs reported changes in the weekly mean WINRS, involving 1055 patients. Based on our meta-analysis results, the following KOR agonists demonstrated significant benefits in relieving the severity of itching compared with placebo: difelikefalin 0.25 µg/kg [SMD =0.58 (95% CI 0.34–0.83), SUCRA 61.0%; high confidence in evidence], followed by difelikefalin 0.5 µg/kg [SMD = 0.45 (95% CI 0.23–0.66), SUCRA 7.0%; high confidence in evidence], difelikefalin 1.5 µg/kg [SMD = 0.41 (95% CI 0.04–0.78), SUCRA 16.0%; high confidence in evidence], difelikefalin 1.0 µg/kg [SMD = 0.37 (95% CI 0.15–0.59)], SUCRA 1.0%; high confidence in evidence] and nalbuphine 120 mg [SMD = 0.30 (95% CI 0.05–0.55), SUCRA 3.0%; high confidence in evidence]. Moreover, difelikefalin 0.25 µg/kg was significantly more effective than nalfurafine 2.5 µg [SMD = −0.45 (95% CI −0.81 to −0.10); high confidence in evidence] and nalbuphine 60 mg [SMD = −0.95 (95% CI −1.85 to −0.05); high confidence in evidence] on alleviating pruritus. All the differences were statistically significant (*P* < .05). Bayesian NMA league tables for each intervention are shown in Table 2, the network plot is shown in Fig. 4A and the forest plot is shown in Supplementary Fig. S1.

**Table 2:** Netleague table (WINRS).

	Placebo	Dif 0.25 μg/kg	Dif 0.5 μg/kg	Dif 1.0 μg/kg	Dif 1.5 μg/kg	Nalbu 120 mg	Nalbu 60 mg	Nalfu 2.5 μg	Nalfu 5 μg	Nalfu 10 μg
Placebo	Placebo	−0.582	−0.447	−0.366	−0.408	−0.3	−0.129	0.367	0.089	−0.012
		(−0.827 to −0.338)	(−0.664 to −0.228)	(−0.585 to −0.146)	(−0.775 to −0.043)	(−0.554 to −0.047)	(−0.383–0.125)	(−0.492–1.23)	(−0.713–0.898)	(−0.852–0.833)
Dif 0.25 μg/kg	**0.582^a^**	Dif 0.25 μg/kg	0.136	0.217	0.175	0.283	0.453	0.949	0.67	0.57
	**(0.338–0.827)**		(−0.112–0.383)	(−0.03–0.464)	(−0.233–0.58)	(−0.072–0.636)	(0.101–0.806)	(0.054–1.845)	(−0.165–1.516)	(−0.304–1.448)
Dif 0.5 μg/kg	**0.447^a^**	−0.136	Dif 0.5 μg/kg	0.081	0.038	0.146	0.317	0.811	0.534	0.434
	**(0.228–0.664)**	(−0.383–0.112)		(−0.142–0.302)	(−0.331–0.406)	(−0.188–0.48)	(−0.019–0.652)	(−0.073–1.705)	(−0.293–1.375)	(−0.432–1.305)
Dif 1.0 μg/kg	**0.366^a^**	−0.217	−0.081	Dif 1.0 μg/kg	−0.043	0.066	0.236	0.732	0.454	0.353
	**(0.146–0.585)**	(−0.464–0.03)	(−0.302–0.142)		(−0.412–0.33)	(−0.269–0.403)	(−0.097–0.57)	(−0.156–1.623)	(−0.372–1.292)	(−0.509–1.225)
Dif 1.5 μg/kg	**0.408^a^**	−0.175	−0.038	0.043	Dif 1.5 μg/kg	0.108	0.279	0.776	0.496	0.396
	**(0.043–0.775)**	(−0.58–0.233)	(−0.406–0.331)	(−0.33–0.412)		(−0.335–0.556)	(−0.166–0.727)	(−0.157–1.714)	(−0.384–1.383)	(−0.517–1.314)
Nalbu 120 mg	**0.3^a^**	−0.283	−0.146	−0.066	−0.108	Nalbu 120 mg	0.17	0.668	0.387	0.288
	**(0.047–0.554)**	(−0.636–0.072)	(−0.48–,0.188)	(−0.403–0.269)	(−0.556–0.335)		(−0.088–0.43)	(−0.229–1.567)	(−0.451–1.228)	(−0.586–1.171)
Nalbu 60 mg	0.129	**−0.453^a^**	−0.317	−0.236	−0.279	−0.17	Nalbu 6 0mg	0.498	0.216	0.117
	(−0.125–0.383)	**(−0.806 to** −**0.101)**	(−0.652–0.019)	(−0.57–0.097)	(−0.727–0.166)	(−0.43–0.088)		(−0.399–1.396)	(−0.624–1.063)	(−0.758–0.998)
Nalfu 2.5 μg	−0.367	**−0.949^a^**	−0.811	−0.732	−0.776	−0.668	−0.498	Nalfu 2.5 μg	−0.28	−0.378
	(−1.23–0.492)	**(−1.845 to −0.054)**	(−1.705–0.073)	(−1.623–0.156)	(−1.714–0.157)	(−1.567–0.229)	(−1.396–0.399)		(−1.113–0.563)	(−1.25–0.493)
Nalfu 5 μg	−0.089	−0.67	−0.534	−0.454	−0.496	−0.387	−0.216	0.28	Nalfu 5 μg	−0.097
	(−0.898–0.713)	(−1.516–0.165)	(−1.375–0.293)	(−1.292–0.372)	(−1.383–0.384)	(−1.228–0.451)	(−1.063–0.624)	(−0.563–1.113)		(−0.918–0.716)
Nalfu 10 μg	0.012	−0.57	−0.434	−0.353	−0.396	−0.288	−0.117	0.378	0.097	Nalfu 10 μg
	(−0.833–0.852)	(−1.448–0.304)	(−1.305–0.432)	(−1.225–0.509)	(−1.314–0.517)	(−1.171–0.586)	(−0.998–0.758)	(−0.493–1.25)	(−0.716–0.918)	

Net league table of head-to-head comparisons for different doses of KOR agonists in pruritus relief under the WINRS.

Significant values in bold.

Dif: difelikefalin; Nalbu: nalbuphine; Nalfu: nalfurafine.

### The VAS

As shown in Table 1, four RCTs reported changes in the VAS, including 12 arms, 6 pairwise comparisons and 574 patients. Upon meta-analysis, nalfurafine 5 μg [SMD = 0.48 (95% CI 0.27–0.69), SUCRA 47.0%; high confidence of evidence] was most effective in mitigating pruritus, followed by nalfurafine 2.5 μg [SMD = 0.46 (95% CI 0.25–0.67), SUCRA 33.0%; high confidence of evidence]. The differences were of statistical significance (*P* < .05). Bayesian NMA league tables for each intervention are shown in Table 3, the network plot is shown in Fig. 4B and the forest plot is shown in Supplementary Fig. S2.

**Table 3:** Netleague table (VAS).

	Placebo	Nalfu 2.5 μg	Nalfu 5 μg	Nalfu 10 μg
Placebo	Placebo	−0.459	−0.48	−0.219
		(−0.67 to −0.245)	(−0.69 to −0.27)	(−0.923–0.485)
Nalfu 2.5 μg	**0.459^a^**	Nalfu 2.5 μg	−0.022	0.24
	**(0.245,0.67)**		(−0.221–0.175)	(−0.464–0.942)
Nalfu 5 μg	**0.480^a^**	0.022	Nalfu 5 μg	0.261
	**(0.27,0.69)**	(−0.175–0.221)		(−0.436–0.962)
Nalfu 10 μg	0.219	−0.24	−0.261	Nalfu 10 μg
	(-0.485,0.923)	(−0.942–0.464)	(−0.962–0.436)	

Net league table of head-to-head comparisons for different doses of KOR agonists in pruritus relief under the VAS.

Nalfu: nalfurafine.

### The 5-D itch scale

As shown in Table 1, six RCTs reported changes in the 5-D itch scale, involving 1564 patients. As revealed by our meta-analysis results, the three doses of difelikefalin demonstrated significant benefits in relieving itching severity relative to placebo. Specifically, difelikefalin 0.25 μg/kg [SMD = 0.33 (95% CI 0.12–0.54), SUCRA 38.0%; high confidence in evidence] was most effective, followed by difelikefalin 0.5 μg/kg [SMD = 0.30 (95% CI 0.18–0.41), SUCRA 17.0%; high confidence in evidence] and difelikefalin 1.0 μg/kg [SMD = 0.25 (95% CI 0.09–0.41), SUCRA 7.0%; high confidence in evidence]. Additionally, these three doses of difelikefalin were more effective than difelikefalin 1.5 μg/kg and all the four doses of difelikefalin and nalfurafine 5 μg were significantly more effective than nalfurafine 2.5 μg in alleviating pruritus. Bayesian NMA league tables for each intervention are presented in Table 4, the network plot is shown in Fig. 4C and the forest plot is shown in Supplementary Fig. S3.

**Table 4:** Netleague table (5-D Itch Scale).

	Placebo	Dif 0.25 μg/kg	Dif 0.5 μg/kg	Dif 1.0 μg/kg	Dif 1.5 μg/kg	Nalfu 2.5 μg	Nalfu 5 μg	Nalfu 10 μg
Placebo	Placebo	−0.331	−0.295	−0.253	−0.061	0.859	−0.22	0.31
		(−0.543 to−0.118)	(−0.406 to −0.184)	(−0.413 to −0.093)	(−0.254–0.132)	(−0.025–1.746)	(−1.023–0.586)	(−0.531–1.158)
Dif 0.25 μg/kg	**0.331^a^**	Dif 0.25 μg/kg	0.036	0.078	0.27	1.189	0.11	0.64
	**(0.118–0.543)**		(−0.171–0.245)	(−0.142–0.298)	(0.015–0.526)	(0.285–2.103)	(−0.719–0.943)	(−0.225–1.514)
Dif 0.5 μg/kg	**0.295^a^**	−0.036	Dif 0.5 μg/kg	0.042	0.234	1.15	0.075	0.604
	**(0.184–0.406)**	(−0.245–0.171)		(−0.098–0.183)	(0.063–0.403)	(0.264–2.05)	(−0.736–0.889)	(−0.243–1.458)
Dif 1.0 μg/kg	**0.253^a^**	−0.078	−0.042	Dif 1.0 μg/kg	0.191	1.11	0.032	0.562
	**(0.093–0.413)**	(−0.298–0.142)	(−0.183–0.098)		(0.019, 0.363)	(0.216–2.016)	(−0.79–0.855)	(−0.293–1.424)
Dif 1.5 μg/kg	0.061	**−0.27^a^**	**−0.234^a^**	**−0.191^a^**	Dif 1.5 μg/kg	0.919	−0.158	0.372
	(−0.132–0.254)	**(−0.526 to −0.015)**	**(−0.403 to −0.063)**	**(−0.363 to −0.019)**		(0.02–1.831)	(−0.984–0.674)	(−0.491–1.242)
Nalfu 2.5 μg	−0.859	**−1.189^a^**	**−1.153^a^**	**−1.11^a^**	**−0.919^a^**	Nalfu 2.5 μg	−1.08	−0.549
	(−1.746–0.025)	**(−2.103 to −0.285)**	**(−2.05 to −0.264)**	**(−2.016 to −0.216)**	**(−1.831 to −0.02)**		(−1.906 to −0.242)	(−1.419–0.323)
Nalfu 5 μg	0.22	−0.11	−0.075	−0.032	0.158	**1.08^a^**	Nalfu 5 μg	0.529
	(−0.586–1.023)	(−0.943–0.719)	(−0.889–0.736)	(−0.855–0.79)	(−0.674–0.984)	**(0.242 to 1.906)**		(−0.257–1.316)
Nalfu 10 μg	−0.31	−0.64	−0.604	−0.562	−0.372	0.549	−0.529	Nalfu 10 μg
	(−1.158–0.531)	(−1.514–0.225)	(−1.458–0.243)	(−1.424–0.293)	(−1.242–0.491)	(−0.323–1.419)	(−1.316–0.257)	

Net league table of head-to-head comparisons for different doses of KOR agonists in pruritus relief under the 5-D Itch Scale.

Significant values in bold.

Dif: difelikefalin; Nalfu: nalfurafine.

### The Skindex-10 scale

As shown in Table 1, seven RCTs reported changes in the Skindex-10 scale, including 1935 patients. NMA showed that difelikefalin 0.5 μg/kg [SMD = 0.23 (95% CI 0.11–0.34), SUCRA 6.0%; high confidence in evidence] was the only novel κ receptor agonist that significantly relieved the severity of itching compared with placebo. Moreover, difelikefalin 0.5 μg/kg showed significant benefit in relieving the severity of itching compared with nalbuphine 60 mg [SMD = −0.31 (95% CI −0.58 to −0.03); high confidence in evidence]. Bayesian NMA league tables for each intervention are shown in Table 5, the network plot is shown in Fig. 4D and the forest plot is shown in Supplementary Fig. S4.

**Table 5:** Netleague table (Skindex-10 Itch Scale).

	Placebo	Dif 0.25 μg/kg	Dif 0.5 μg/kg	Dif 1.0 μg/kg	Dif 1.5 μg/kg	Nalbu 120 mg	Nalbu 60 mg	Nalfu 2.5 μg	Nalfu 5 μg	Nalfu 10 μg
Placebo	Placebo	−0.18	−0.225	−0.085	−0.185	−0.139	0.081	0.319	−0.522	−0.213
		(−0.393–0.033)	(−0.337–−0.112)	(−0.281–0.113)	(−0.535–0.163)	(−0.395–0.115)	(−0.174–0.335)	(−0.543–1.178)	(−1.321–0.283)	(−1.053–0.632)
Dif 0.25 μg/kg	0.18	Dif 0.25 μg/kg	−0.045	0.095	−0.005	0.04	0.26	0.501	−0.343	−0.032
	(−0.033–0.393)		(−0.259–0.17)	(−0.139–0.331)	(−0.398–0.386)	(−0.293–0.375)	(−0.071–0.594)	(−0.39–1.387)	(−1.172–0.493)	(−0.903–0.836)
Dif 0.5 μg/kg	**0.225^a^**	0.045	Dif 0.5 μg/kg	0.14	0.039	0.085	0.306	0.545	−0.297	0.012
	**(0.112–0.337)**	(−0.17–0.259)		(−0.058–0.338)	(−0.309–0.389)	(−0.193–0.365)	(0.029–0.584)	(−0.323–1.411)	(−1.103–0.516)	(−0.837–0.862)
Dif 1.0 μg/kg	0.085	−0.095	−0.14	Dif 1.0 μg/kg	−0.101	−0.056	0.166	0.406	−0.437	−0.13
	(−0.113–0.281)	(−0.331–0.139)	(−0.338–0.058)		(−0.463–0.259)	(−0.377–0.268)	(−0.155–0.486)	(−0.482–1.291)	(−1.265–0.391)	(−0.992–0.742)
Dif 1.5 μg/kg	0.185	0.005	−0.039	0.101	Dif 1.5 μg/kg	0.046	0.266	0.506	−0.337	−0.029
	(−0.163–0.535)	(−0.386–0.398)	(−0.389–0.309)	(−0.259–0.463)		(−0.386–0.476)	(−0.165–0.7)	(−0.429–1.434)	(−1.212–0.543)	(−0.933–0.883)
Nalbu 120 mg	0.139	−0.04	−0.085	0.056	−0.046	Nalbu 120 mg	0.221	0.459	−0.383	−0.073
	(−0.115–0.395)	(−0.375–0.293)	(−0.365–0.193)	(−0.268–0.377)	(−0.476–0.386)		(−0.04–0.48)	(−0.441–1.357)	(−1.224–0.463)	(−0.956–0.807)
Nalbu 60 mg	−0.081	−0.26	**−0.306^a^**	−0.166	−0.266	−0.221	Nalbu 60 mg	0.239	−0.603	−0.295
	(−0.335–0.174)	(−0.594–0.071)	**(−0.584 to −0.029)**	(−0.486–0.155)	(−0.7–0.165)	(−0.48–0.04)		(−0.664–1.136)	(−1.44–0.242)	(−1.175–0.585)
Nalfu 2.5 μg	−0.319	−0.501	−0.545	−0.406	−0.506	−0.459	−0.239	Nalfu 2.5 μg	−0.841	−0.531
	(−1.178–0.543)	(−1.387–0.39)	(−1.411–0.323)	(−1.291–0.482)	(−1.434–0.429)	(−1.357–0.441)	(−1.136–0.664)		(−1.679–0)	(−1.408–0.342)
Nalfu 5 μg	0.522	0.343	0.297	0.437	0.337	0.383	0.603	0.841	Nalfu 5 μg	0.309
	(−0.283–1.321)	(−0.493–1.172)	(−0.516–1.103)	(−0.391–1.265)	(−0.543–1.212)	(−0.463–1.224)	(−0.242–1.44)	(0–1.679)		(−0.505–1.123)
Nalfu 10 μg	0.213	0.032	−0.012	0.13	0.029	0.073	0.295	0.531	−0.309	Nalfu 10 μg
	(−0.632–1.053)	(−0.836–0.903)	(−0.862–0.837)	(−0.742–0.992)	(−0.883–0.933)	(−0.807–0.956)	(−0.585–1.175)	(−0.342–1.408)	(−1.123–0.505)	

Net league table of head-to-head comparisons for different doses of KOR agonists in pruritus relief under the Skindex-10 Itch Scale.

Significant values in bold.

Dif: difelikefalin; Nalbu: nalbuphine; Nalfu: nalfurafine.

### Safety

As shown in Table 1, nine RCTs reported adverse events involving 2112 patients. The NMA results indicated that compared with difelikefalin 1.0 μg/kg, nalfurafine 2.5 μg was associated with a significantly lower incidence of adverse events [SMD = 1.28 (95% CI 1.02–1.60)]. In contrast, the four doses of difelikefalin were associated with higher adverse event rates than placebo: 0.25 μg/kg [SMD = 0.85 (95% CI 0.73–0.99)], 0.5 μg/kg [SMD = 0.84 (95% CI 0.76–0.93)], 1.0 μg/kg [SMD = 0.79 (95% CI 0.69–0.91)] and 1.5 μg/kg [SMD = 0.76 (95% CI 0.62–0.96)]. It appeared that the higher doses were associated with a greater risk. Moreover, the incidence rates of adverse events of other interventions were comparable to that of the placebo and there was no statistically significant difference. Among the interventions, difelikefalin was most commonly associated with side effects, including loose bowel movements, dizziness, nausea, increased drowsiness and a propensity for falls. Nalfurafine was frequently reported to disrupt sleep patterns, while gastrointestinal disturbances were primarily linked to nalbuphine. Based on the available data, most adverse reactions were mild to moderate and treatment discontinuation was rarely necessary. Bayesian NMA league tables for each intervention are presented in Table 6 and the network plot is shown in Fig. 4E.

**Table 6:** Netleague table (adverse events).

	Placebo	Dif 0.25 μg/kg	Dif 0.5 μg/kg	Dif 1.0 μg/kg	Dif 1.5 μg/kg	Nalfu 2.5 μg	Nalfu 5 μg	Nalfu 10 μg
Placebo	Placebo	1.183	1.192	1.268	1.32	0.988	1.06	1.602
		(1.01–1.376)	(1.074–1.325)	(1.105–1.446)	(1.039–1.626)	(0.832–1.181)	(0.91–1.253)	(0.937–2.567)
Dif 0.25 μg/kg	**0.845^a^**	Dif 0.25 μg/kg	1.007	1.071	1.115	0.836	0.898	1.354
	**(0.727–0.99)**		(0.875–1.171)	(0.925–1.246)	(0.862–1.418)	(0.664–1.061)	(0.722–1.128)	(0.778–2.23)
Dif 0.5 μg/kg	**0.839^a^**	0.993	Dif 0.5 μg/kg	1.063	1.107	0.829	0.889	1.342
	**(0.755–0.931)**	(0.854–1.143)		(0.935–1.205)	(0.873–1.368)	(0.677–1.02)	(0.737–1.084)	(0.78–2.179)
Dif 1.0 μg/kg	**0.789^a^**	0.933	0.941	Dif 1.0 μg/kg	1.042	0.78	0.837	1.263
	**(0.692–0.905)**	(0.803–1.081)	(0.83–1.07)		(0.826–1.282)	(0.626–0.976)	(0.682–1.039)	(0.728–2.067)
Dif 1.5 μg/kg	**0.758^a^**	0.896	0.903	0.96	Dif 1.5 μg/kg	0.749	0.805	1.215
	**(0.615–0.962)**	(0.705–1.16)	(0.731–1.146)	(0.78–1.211)		(0.57–1.007)	(0.617–1.073)	(0.685–2.056)
Nalfu 2.5 μg	1.012	1.196	1.207	**1.282^a^**	1.335	Nalfu 2.5 μg	1.073	1.619
	(0.847–1.202)	(0.943–1.507)	(0.981–1.477)	**(1.024–1.597)**	(0.993–1.755)		(0.932–1.247)	(0.951–2.611)
Nalfu 5 μg	0.943	1.114	1.124	1.195	1.242	0.932	Nalfu 5 μg	1.506
	(0.798–1.099)	(0.886–1.385)	(0.922–1.356)	(0.962–1.466)	(0.932–1.62)	(0.802–1.073)		(0.889–2.401)
Nalfu 10 μg	0.624	0.739	0.745	0.792	0.823	0.618	0.664	Nalfu 10 μg
	(0.389–1.067)	(0.448–1.286)	(0.459–1.282)	(0.484–1.373)	(0.486–1.459)	(0.383–1.052)	(0.417–1.124)	

Net league table of head-to-head comparisons for different doses of KOR agonists in the incidence of adverse events.

Significant values in bold.

## DISCUSSION

### Principal findings

KOR agonists have been extensively investigated for their potential in treating various centrally mediated conditions, including pruritus. These agonists exert antipruritic effects by binding to KORs located on keratinocytes, skin and central neuronal populations involved in itch signal processing. As non-addictive analgesic agents that do not induce respiratory depression, KOR agonists have garnered significant interest in both clinical and research settings [38, 39]. This distinctive pharmacological profile arises from KOR agonists’ selective engagement of KORs, which are anatomically and functionally distinct from μ-opioid receptors implicated in euphoria and addiction [40]. Crucially, KOR agonists preferentially activate G protein-coupled signalling pathways, which dissociates analgesic efficacy from addictive potential through differential neural circuitry engagement [41]. In our meta-analysis, a network approach was adopted to integrate both direct and indirect estimates of difelikefalin, nalfurafine and nalbuphine to assess their efficacy and safety. Our preliminary findings indicated that difelikefalin at doses of 0.25, 0.5, 1.0 and 1.5 µg/kg, nalfurafine at 2.5 and 5 µg and nalbuphine at 120 mg were significantly effective in relieving itching severity compared with placebo. Specifically, difelikefalin at 0.25 and 0.5 µg/kg showed significantly greater efficacy than nalbuphine at 60 mg in alleviating pruritus. Additionally, difelikefalin at 0.25, 0.5 and 1.0 µg/kg was more effective than difelikefalin at 1.5 µg/kg, and all the four doses of difelikefalin and nalfurafine at 5 µg were significantly more effective than nalfurafine at 2.5 µg in alleviating pruritus. No significant differences were observed in itching relief among other comparisons or between treatment and placebo groups.

Based on the results of NMA and cumulative probability ranking, it was concluded that difelikefalin at doses of 0.25 and 0.5 µg/kg was most effective, with no significant difference in the incidence of adverse effects between these two doses. Nalfurafine at doses of 5 and 2.5 µg also demonstrated significant efficacy in reducing pruritus and exhibited a better safety profile than difelikefalin, making it a viable alternative, while nalfurafine at 5 µg provided superior pruritus relief to the 2.5-µg dose. However, the lack of direct comparisons with difelikefalin prevented a definitive conclusion that KOR agonist was more effective in pruritus relief. Therefore, further head-to-head trials are warranted to confirm these findings.

Nalfurafine, which has not yet received US Food and Drug Administration (FDA) approval, has been approved mainly in Asian markets. Japan was the first to approve its use for HD-related uraemic pruritus in 2009, and in 2017 its indication was expanded to peritoneal dialysis–related pruritus. In July 2023, China approved nalfurafine hydrochloride orally dispersible tablets, making it the first κ receptor agonist (as an antipruritic agent) approved in the country [42, 43]. NMAs have consistently demonstrated that nalfurafine at doses of both 0.5 and 0.25 µg is effective in reducing itching severity, aligning with previous research findings [42]. According to our results, it seemed that nalfurafine at 5 µg offered greater efficacy than the 2.5-µg dose [SMD = 1.08 (95% CI 0.24–1.90)], while the incidence of adverse reactions remained comparable between the two doses [SMD = 0.93 (95% CI 0.80–1.07)]. A multiple ascending dose study found hypotension, dizziness and lower abdominal pain as the common treatment-related adverse events [44]. A post-marketing surveillance study recruiting 3762 HD patients with refractory itching reported that oral nalfurafine hydrochloride (2.5–5.0 µg/day) was both safe and effective. The common drug-related adverse events included insomnia, constipation, somnolence, dizziness, nausea and asthenia. Approximately 80% of patients showed significant symptom improvement at 12 weeks and 1 year, and the adverse reaction rates were similar between the 2.5-µg and 5-µg doses [43]. An international multicentre study examined the possibility of abuse liability of nalfurafine in 146 HD patients, indicating no evidence of abuse liability [45]. A literature review indicated that adverse reactions to nalfurafine are generally mild to moderate, with insomnia being the most commonly reported side effect. Due to the transient and manageable nature of these reactions, nalfurafine is considered a safe therapeutic option.

In contrast, difelikefalin is a selective, peripherally acting KOR agonist that targets primarily peripheral neurons and immune system cells. In August 2021, intravenous administration of difelikefalin was approved in the USA for the treatment of moderate to severe pruritus in adult patients with CKD undergoing HD. Subsequently, in April 2022, the drug received approval from the European Medicines Agency and has been used in Switzerland, Canada, Singapore, Australia and various other countries [46–49]. Five RCTs evaluated the efficacy and safety of difelikefalin (0.25, 0.5, 1.0 and 1.5 μg/kg) versus control groups in alleviating UP. The results showed that difelikefalin was effective across all doses, with 0.25-, 0.5- and 1.0-μg/kg doses demonstrating superior efficacy to the 1.5-μg/kg dose. This NMA also indicated that adverse reactions with difelikefalin were more frequent than those with placebo, which increased in a dose-dependent manner, consistent with previous findings [31, 33]. However, most adverse reactions were mild, including diarrhoea, dizziness, nausea, somnolence and falls, which occurred early in treatment and rarely required discontinuation [32]. No dose-related cumulative reactions affecting efficacy were observed. Overall, difelikefalin at doses of 0.25 and 0.5 μg/kg significantly improves moderate to severe pruritus and has a high safety profile and thus these doses are recommended for clinical use.

Nalbuphine is an FDA-approved mixed μ-opioid receptor antagonist and κ-opioid receptor agonist that is currently marketed for the treatment of moderate to severe pain in patients requiring opioids where other alternative therapies have proven ineffective. Its new indications, such as UP, are still under investigation [50]. Only one RCT assessed the efficacy and safety of nalbuphine (120 mg and 60 mg) versus control groups in relieving pruritus in UP patients. The results indicated that nalbuphine at 120 mg was significantly more effective than placebo in reducing pruritus, as measured by the NRS. In addition, an open-label, multiple ascending dose study found that nalbuphine extended-release tablets effectively reduced itching severity in HD patients, with a notable effect at doses of ≥60 mg twice a day [50]. Also, the symptom severity decreased in a dose-dependent manner. Despite the small sample size, the study suggested that nalbuphine could be safely administered at a dose up to 240 mg twice a day. Previous studies indicated that serious adverse events were rare with nalbuphine and the most common treatment-related adverse reactions were gastrointestinal and neurological adverse reactions, consistent with opioid-related drugs [51].

### Limitations

This study represents the first NMA comparing the efficacy and safety of currently available KOR agonists for the treatment of UP, but it has certain limitations. First, the included trials varied in their follow-up periods, and most RCTs had a follow-up period of <1 year. Due to the short follow-up period, itching may recur, but our included RCTs did not report the recurrence rate, which might lead to imprecise effect estimates. Second, given the nascent stage of KOR agonists as a novel therapeutic intervention, they have not been extensively commercialized or widely adopted in clinical practice globally. Consequently, the number of RCTs available for inclusion in our NMA was limited and direct head-to-head trials are lacking. We acknowledge that the limited number of studies and comparisons might introduce potential bias, which may lead to a high risk of bias in the estimation of its effects and instability in the network structure, especially in the comparison of nalbuphine based on only one RCT.

To minimize these limitations, future high-quality RCTs on KOR agonists, particularly head-to-head comparative trials, are strongly recommended to refine network evidence and validate current findings. Additionally, when designing future trials, sample sizes should be increased to improve the robustness of the findings. Longer follow-up periods should be considered to capture the potential recurrence of symptoms and to provide a more comprehensive understanding of the long-term benefits and risks associated with KOR agonist therapy. In this way, more definitive conclusions about the efficacy and safety of KOR agonists for the treatment of pruritus associated with chronic renal failure will be provided.

## CONCLUSION

The present study suggests that difelikefalin at doses of 0.25 and 0.5 µg/kg, along with nalfurafine at doses of 0.25 and 0.5 µg/kg, can be considered as recommended therapeutic options for UP treatment. However, future prospective, multicentre and head-to-head RCTs are needed to further validate these findings.

## Supplementary Material

sfaf131_Supplemental_Files

## Data Availability

Full extracted data from included studies can be requested from the corresponding author.
