# Supplementary material for: The efficacy and safety of kappa opioid receptor (KOR) agonists in patients with uraemic pruritus: a systematic review and network meta-analysis
Source: Clin Kidney J. 2025 Jun 23;18(6):sfaf131. doi: 10.1093/ckj/sfaf131 (PMC12188196; doi:10.1093/ckj/sfaf131)
Supplement: sfaf131_Supplemental_Files [file sfaf131_supplemental_files.zip › Supplementary Table.docx]

**TableS1. Systematic search strategies**

| **Database** | **Search strategy** | **Result** |
| --- | --- | --- |
| **PubMed**  Results = 48 | #1. “Uremia”[Title/Abstract] OR “Uremias” [Title/Abstract] OR "Uremia"[Mesh]  #2. “Renal Failure, Chronic” [Title/Abstract] OR “Chronic Renal Failure” [Title/Abstract] OR “End-Stage Kidney Disease” [Title/Abstract]OR “Disease, End-Stage Kidney” [Title/Abstract] OR “End Stage Kidney Disease” [Title/Abstract] OR “Kidney Disease, End-Stage ESRD” [Title/Abstract] OR “End-Stage Renal Disease” [Title/Abstract] OR “Disease, End-Stage Renal” [Title/Abstract]OR “End Stage Renal Disease” [Title/Abstract] OR “Renal Disease, End-Stage” [Title/Abstract] OR “Renal Disease, End Stage” [Title/Abstract] OR “Renal Failure, End-Stage” [Title/Abstract] OR “End-Stage Renal Failure” [Title/Abstract] OR “Renal Failure, End Stage” [Title/Abstract] OR “Chronic Kidney Failure” [Mesh]  #3.“Dialyses, Renal”[Title/Abstract] OR “Renal Dialyses”[Title/Abstract] OR “Dialysis, Renal”[Title/Abstract] OR “Hemodialysis”[Title/Abstract] OR “Hemodialyses”[Title/Abstract] OR “Dialysis, Extracorporeal”[Title/Abstract] OR “Dialyses, Extracorporeal”[Title/Abstract] OR “Extracorporeal Dialyses”[Title/Abstract] OR “Extracorporeal Dialysis”[Title/Abstract] OR“Renal Dialysis”[Mesh]  #4. #1 OR #2 OR #3  #5. Puritus[Title/Abstract] OR Pruritis[Title/Abstract] OR Itching[Title/Abstract] OR"Pruritus"[Mesh]  #6“Opioid Receptors, kappa” [Title/Abstract] OR “kappa Opioid Receptors” [Title/Abstract] OR“Receptors, kappa Opioid” [Title/Abstract] OR“kappa Opioid Receptor” [Title/Abstract] OR“Opioid Receptor, kappa” [Title/Abstract] OR“Receptor, kappa Opioid” [Title/Abstract] OR“kappa Receptor” [Title/Abstract] OR“Receptor, kappa” [Title/Abstract] OR“kappa Receptors” [Title/Abstract] OR“Receptors, kappa” [Title/Abstract]OR"Receptors, Opioid, kappa"[Mesh]  #7 #4AND#5AND#6 | 29,940  139,665  152,075  246,696  26,145  7,750  48 |
| **Embase**  Results = 109 | #1 'uremia'/exp OR 'azotaemia' OR 'azotemia' OR 'hyperazotemia' OR 'hyperuraemia' OR 'hyperuremia' OR 'uraemia' OR 'uraemic serum' OR 'uraemic syndrome' OR 'uremic serum' OR 'uremic syndrome' OR 'uremia'  #2 'renal failure'/exp OR'kidney insufficiency' OR 'maternal kidney failure' OR 'renal failure' OR 'renal insufficiency' OR 'terminal kidney failure' OR 'kidney failure'  #3 'renal replacement therapy'/exp OR 'dialysis therapy' OR 'dialysis treatment' OR 'kidney dialysis' OR 'kidney replacement therapy' OR 'kidney support' OR 'renal support' OR 'renal replacement therapy'  #4 #1 OR #2 OR #3  #5 'itch'/exp OR 'itch' OR 'itching'/exp OR 'itching' OR 'itchy sensation'/exp OR 'itchy sensation' OR 'pruritic disease'/exp OR 'pruritic disease' OR 'pruritic disorder'/exp OR 'pruritic disorder' OR 'pruritis'/exp OR 'pruritis' OR 'pruritus'/exp OR 'pruritus'  #6 'kappa opiate receptor'/exp OR 'kappa opioid receptor' OR 'kappa receptor' OR 'opiate kappa receptor' OR 'opioid kappa receptor' OR 'opioid receptor kappa' OR 'receptors, opioid, kappa' OR 'kappa opiate receptor'  #7 #4 AND #5 AND #6 | 56,604  691,469  265,088  814,693  146,969  9,286  109 |
| **Cochrane Central Register of Controlled Trials**  Results = 33 | #1 MeSH descriptor: [Uremia] explode all trees  #2 (Uremia):ti,ab,kw OR (Uremias):ti,ab,kw  #3 #1 OR #2  #4 MeSH descriptor: [Kidney Failure, Chronic] explode all trees  #5 (Disease, End-Stage Renal):ti,ab,kw OR (End Stage Kidney Disease):ti,ab,kw OR ( End Stage Renal Disease):ti,ab,kw OR ( Chronic Kidney Failure):ti,ab,kw OR ( Renal Disease, End Stage):ti,ab,kw OR ( Kidney Disease, End-Stage):ti,ab,kw OR ( End-Stage Renal Disease):ti,ab,kw OR ( Renal Failure, Chronic):ti,ab,kw OR ( Chronic Renal Failure):ti,ab,kw OR (ESRD):ti,ab,kw OR ( Renal Disease, End-Stage):ti,ab,kw OR ( End-Stage Kidney Disease):ti,ab,kw OR ( Renal Failure, End Stage):ti,ab,kw OR ( Disease, End-Stage Kidney):ti,ab,kw OR (End-Stage Renal Failure):ti,ab,kw OR ( Renal Failure, End-Stage):ti,ab,kw  #6 #1 OR #2  #7 MeSH descriptor: [Renal Dialysis] explode all trees  #8 (Dialysis, Extracorporeal):ti,ab,kw OR ( Extracorporeal Dialysis):ti,ab,kw OR ( Extracorporeal Dialyses):ti,ab,kw OR ( Dialyses, Extracorporeal):ti,ab,kw OR ( Renal Dialyses):ti,ab,kw OR ( Dialyses, Renal):ti,ab,kw OR ( Dialysis, Renal):ti,ab,kw OR ( Hemodialysis;):ti,ab,kw OR (Hemodialyses):ti,ab,kw  #9 #7 OR #8  #10 #3 OR #6 OR #9  #11 MeSH descriptor: [Pruritus] explode all trees  #12 (Pruritus):ti,ab,kw OR ( Pruritis):ti,ab,kw  #13 #11 OR #12  #14 MeSH descriptor: [Receptors, Opioid, kappa] explode all trees  #15 (Receptors, kappa Opioid):ti,ab,kw OR ( kappa Receptors):ti,ab,kw OR (kappa Opioid Receptor):ti,ab,kw OR ( Receptor, kappa):ti,ab,kw OR ( Opioid Receptor, kappa):ti,ab,kw OR ( Receptors, kappa):ti,ab,kw OR ( Opioid Receptors, kappa):ti,ab,kw OR ( Receptor, kappa Opioid):ti,ab,kw OR ( kappa Opioid Receptors):ti,ab,kw OR ( kappa Receptor):ti,ab,kw  #16 #14 OR #15  #17 #10 AND #13 AND #16 | 573  1,087  1,149  6096  21,404  21,404  7,427  20,273  20,903  33,827  2,079  16,142  16,336  90  757  757  33 |
| **Web of Science**  Results = 12 | #1 Uremia (Topic)or uremics (Topic)or Renal Failure, Chronic (Topic)or Chronic Renal Failure (Topic)or End-Stage Kidney Disease (Topic)or Disease, End-Stage Kidney (Topic)or End Stage Kidney Disease (Topic)or Kidney Disease, End-Stage ESRD (Topic)or End-Stage Renal Disease (Topic)or Disease, End-Stage Renal (Topic)or End Stage Renal Disease (Topic)or Renal Disease, End-Stage (Topic)or Renal Disease, End Stage (Topic)or Renal Failure, End-Stage (Topic)or End-Stage Renal Failure (Topic)or Renal Failure, End Stage (Topic)or Chronic Kidney Failure (Topic)or Renal dialysis (Topic)or Dialysis, Renal (Topic)or hemodialysis (Topic)or hemodialyses (Topic)or Dialysis, Extracorporeal (Topic)or dialysis, Extracorporeal (Topic)or Extracorporeal dialysis (Topic)or Extracorporeal Dialysis (Topic)  #2 Pruritus (Topic)or Pruritis(Topic)or Itching(Topic)  #3 Receptors, kappa Opioid (Topic)or kappa Receptors (Topic)or kappa Opioid Receptor (Topic)or Receptor, kappa (Topic)or Opioid Receptor, kappa (Topic)or Receptors, kappa (Topic)or Opioid Receptors, kappa (Topic)or Receptor, kappa Opioid (Topic)or kappa Opioid Receptors (Topic)or kappa Receptor (Topic)  #4 #1 AND #2 AND #3 | 568,390  79,147  3,470  12 |

**TableS2**. GRADE for the Outcomes.

| **Outcomes/Interventions** | | **Study limitations** | **Inconsistency of results** | **Indirectness of evidence** | **Imprecision** | **Reporting bias** | **Certainty** |
| --- | --- | --- | --- | --- | --- | --- | --- |
| **WINRS** | **Difelikefalin 0.25μg/kg** | **None** | **None** | **None** | **None** | **None** | **High** |
|  | **Difelikefalin 0.5μg/kg** | **Not serious** | **None** | **None** | **None** | **None** | **High** |
|  | **Difelikefalin 1.0μg/kg** | **None** | **None** | **None** | **None** | **None** | **High** |
|  | **Difelikefalin 1.5μg/kg** | **None** | **None** | **None** | **serious** | **None** | **Moderate** |
|  | **Nalbuphine 120mg** | **Not serious** | **None** | **None** | **Not serious** | **None** | **Moderate** |
|  | **Nalbuphine 60mg** | **Not serious** | **None** | **None** | **Not serious** | **None** | **Moderate** |
|  | **Nalfurafine 2.5 μg** | **Not serious** | **None** | **None** | **Not serious** | **None** | **Moderate** |
|  | **Nalfurafine 5 μg** | **Not serious** | **None** | **None** | **Not serious** | **None** | **Moderate** |
|  | **Nalfurafine 10μg** | **None** | **None** | **None** | **serious** | **None** | **Moderate** |
| **VAS** | **Nalfurafine 2.5 μg** | **Not serious** | **None** | **None** | **Not serious** | **None** | **Moderate** |
|  | **Nalfurafine 5 μg** | **Not serious** | **None** | **None** | **Not serious** | **None** | **Moderate** |
|  | **Nalfurafine 10μg** | **Not serious** | **None** | **None** | **serious** | **None** | **Low** |
| **5D** | **Difelikefalin 0.25μg/kg** | **None** | **None** | **None** | **None** | **None** | **High** |
|  | **Difelikefalin 0.5μg/kg** | **Not serious** | **None** | **None** | **None** | **None** | **High** |
|  | **Difelikefalin 1.0μg/kg** | **None** | **None** | **None** | **None** | **None** | **High** |
|  | **Difelikefalin 1.5μg/kg** | **None** | **None** | **None** | **serious** | **None** | **Moderate** |
|  | **Nalfurafine 2.5 μg** | **Not serious** | **None** | **None** | **Not serious** | **None** | **Moderate** |
|  | **Nalfurafine 5 μg** | **Not serious** | **None** | **None** | **Not serious** | **None** | **Moderate** |
|  | **Nalfurafine 10μg** | **None** | **None** | **None** | **serious** | **None** | **Moderate** |
| **S-10** | **Difelikefalin 0.25μg/kg** | **None** | **None** | **None** | **None** | **None** | **High** |
|  | **Difelikefalin 0.5μg/kg** | **Not serious** | **None** | **None** | **None** | **None** | **High** |
|  | **Difelikefalin 1.0μg/kg** | **None** | **None** | **None** | **None** | **None** | **High** |
|  | **Difelikefalin 1.5μg/kg** | **None** | **None** | **None** | **serious** | **None** | **Moderate** |
|  | **Nalbuphine 120mg** | **Not serious** | **None** | **None** | **Not serious** | **None** | **Moderate** |
|  | **Nalbuphine 60mg** | **Not serious** | **None** | **None** | **Not serious** | **None** | **Moderate** |
|  | **Nalfurafine 2.5 μg** | **Not serious** | **None** | **None** | **Not serious** | **None** | **Moderate** |
|  | **Nalfurafine 5 μg** | **Not serious** | **None** | **None** | **Not serious** | **None** | **Moderate** |
|  | **Nalfurafine 10μg** | **None** | **None** | **None** | **serious** | **None** | **High** |
| **WINRS** | **Difelikefalin 0.25μg/kg** | **None** | **None** | **None** | **None** | **None** | **High** |
|  | **Difelikefalin 0.5μg/kg** | **Not serious** | **None** | **None** | **None** | **None** | **High** |
|  | **Difelikefalin 1.0μg/kg** | **None** | **None** | **None** | **None** | **None** | **High** |
|  | **Difelikefalin 1.5μg/kg** | **None** | **None** | **None** | **serious** | **None** | **Moderate** |
|  | **Nalbuphine 120mg** | **Not serious** | **None** | **None** | **Not serious** | **None** | **Moderate** |
|  | **Nalbuphine 60mg** | **Not serious** | **None** | **None** | **Not serious** | **None** | **Moderate** |
|  | **Nalfurafine 2.5 μg** | **Not serious** | **None** | **None** | **Not serious** | **None** | **Moderate** |
|  | **Nalfurafine 5 μg** | **Not serious** | **None** | **None** | **Not serious** | **None** | **Moderate** |
|  | **Nalfurafine 10μg** | **None** | **None** | **None** | **serious** | **None** | **Moderate** |

**We assessed the certainty of evidence using the grading of recommendations assessment, development, and evaluation (GRADE) framework, which characterizes the quality of a body of evidence on the basis of the study limitations, imprecision, inconsistency, indirectness and publication bias for the primary outcomes.**
