# Supplementary figures and images for: The efficacy and safety of kappa opioid receptor (KOR) agonists in patients with uraemic pruritus: a systematic review and network meta-analysis
Source: Clin Kidney J. 2025 Jun 23;18(6):sfaf131. doi: 10.1093/ckj/sfaf131 (PMC12188196; doi:10.1093/ckj/sfaf131)

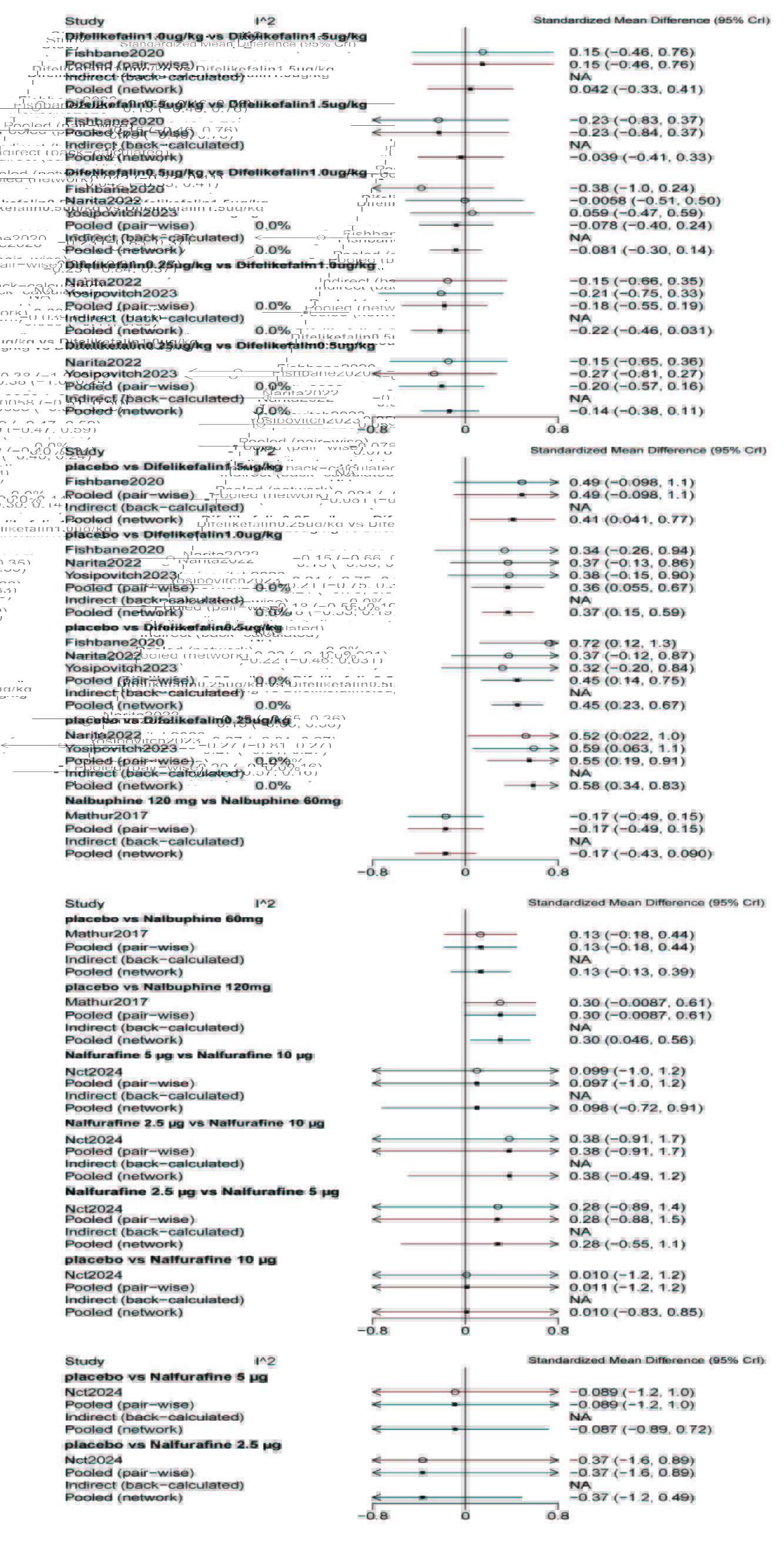

Supplement: sfaf131_Supplemental_Files [file sfaf131_supplemental_files.zip › FigureS1.jpg]

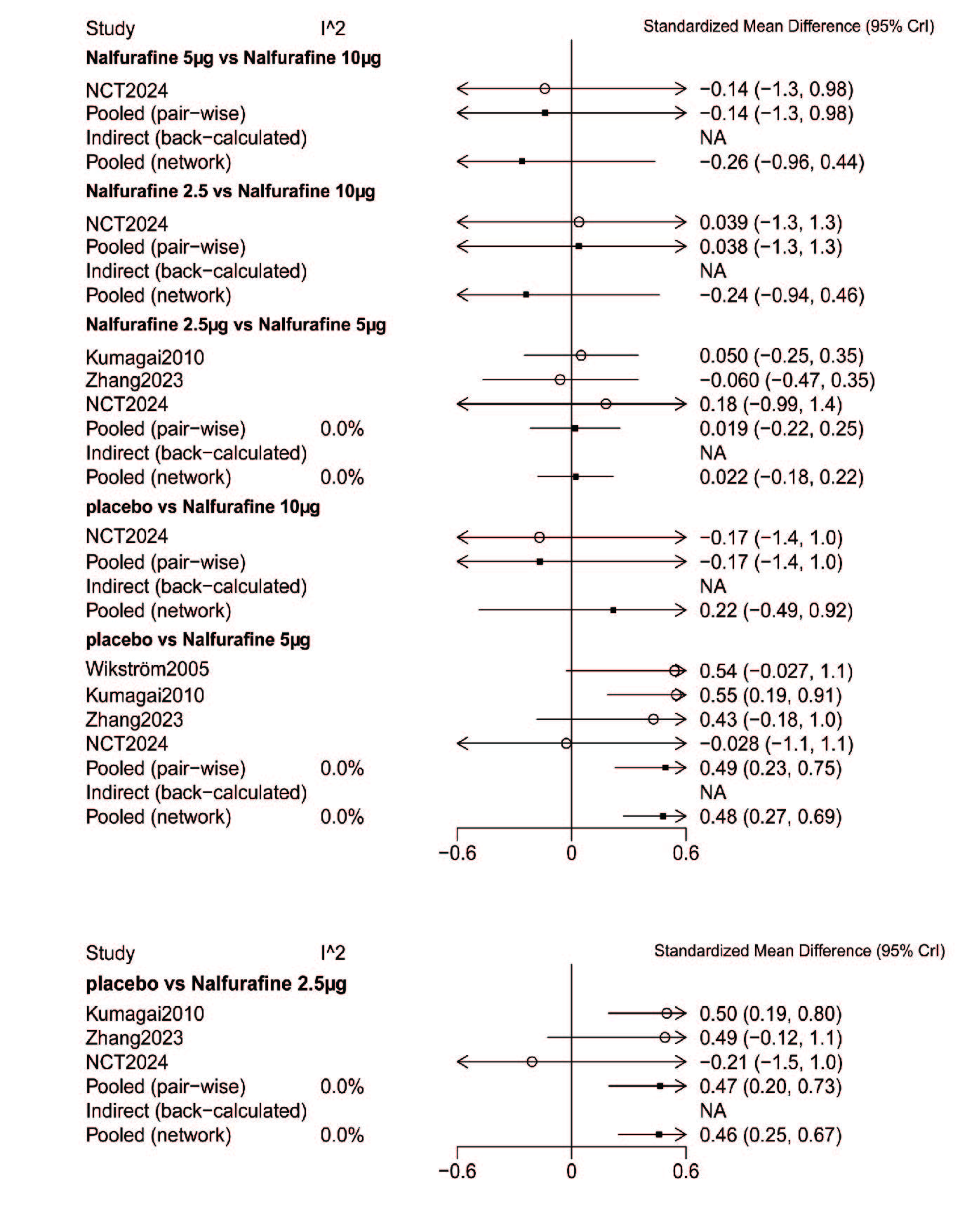

Supplement: sfaf131_Supplemental_Files [file sfaf131_supplemental_files.zip › FigureS2.jpg]

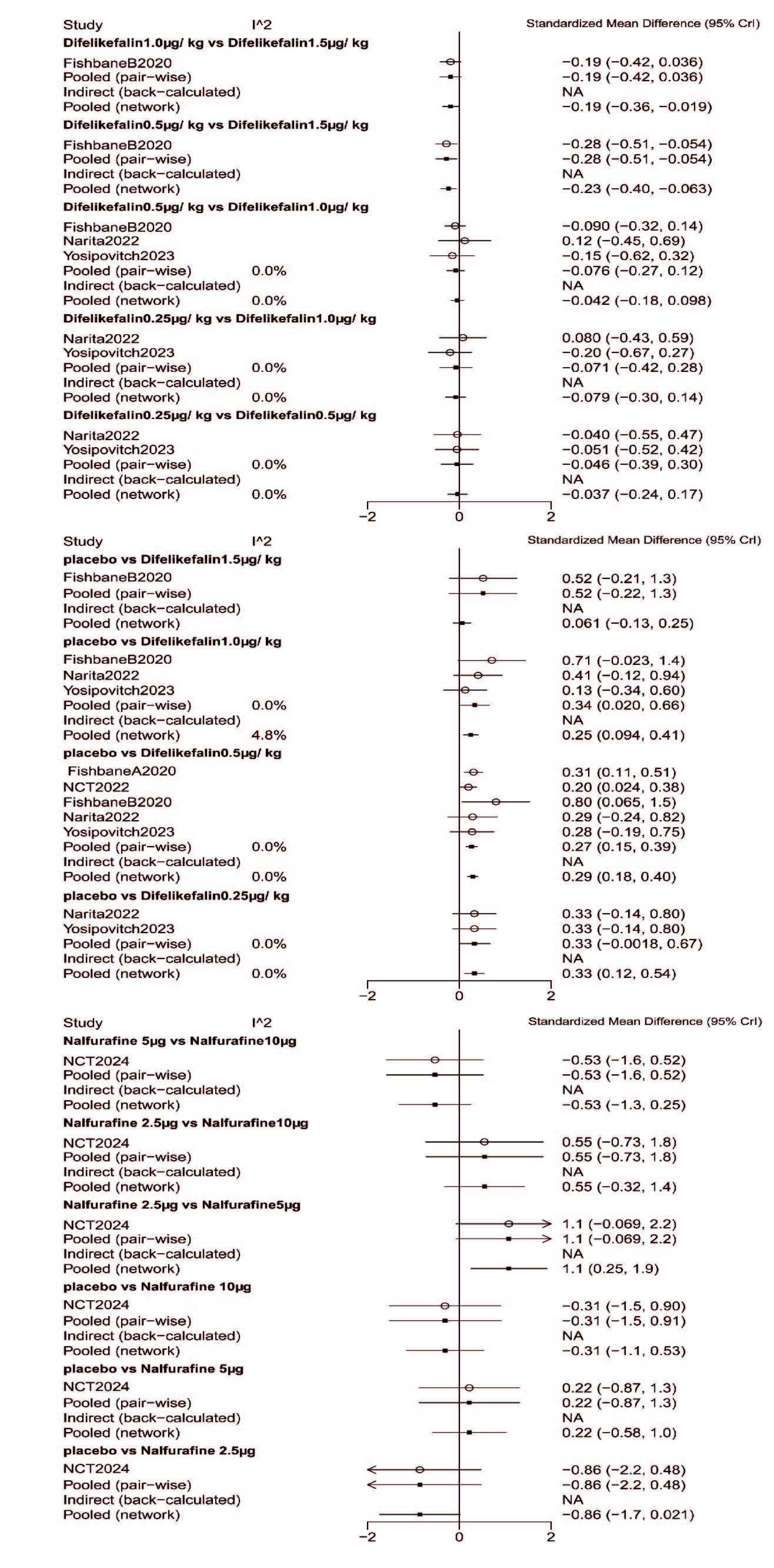

Supplement: sfaf131_Supplemental_Files [file sfaf131_supplemental_files.zip › FigureS3.jpg]

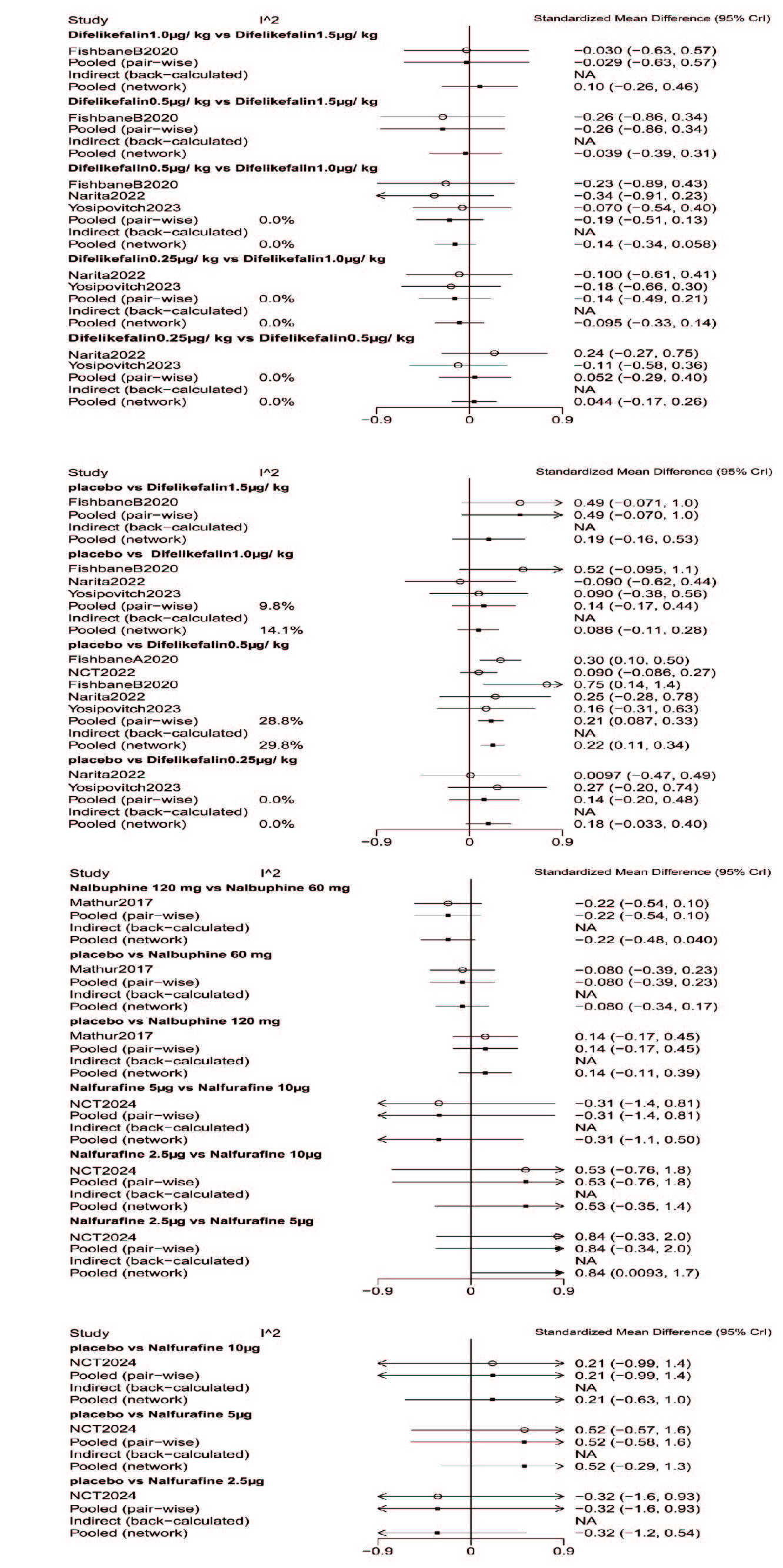

Supplement: sfaf131_Supplemental_Files [file sfaf131_supplemental_files.zip › FigureS4.jpg]
